# Nonconservative vs. Conservative Management of Antenatally Diagnosed Placenta Accreta Spectrum Disorders: A Literature Review of Approaches and Outcomes

**DOI:** 10.1155/ogi/6344708

**Published:** 2025-12-30

**Authors:** Tal Drozdovsky, Man Ho Kwok, Elena Greco

**Affiliations:** ^1^ Faculty of Medicine and Dentistry, Queen Mary University of London, London, UK, qmul.ac.uk; ^2^ Department of Obstetrics and Gynaecology, Royal London Hospital, Barts Health NHS Trust, London, UK, bartshealth.nhs.uk

## Abstract

Placenta accreta spectrum (PAS) disorders involve abnormal placental invasion into the uterine wall and neighbouring organs, posing life‐threatening risks during and after labour. Catastrophic obstetric haemorrhage remains the main morbidity factor, potentially leading to severe complications, including coagulopathy, acute respiratory distress, cardiac arrest, and, in some cases, death. The traditional, nonconservative approach, caesarean hysterectomy, is widely adopted for managing PAS, but it permanently eliminates fertility and carries a high risk of complications. In response, conservative management methods, such as the expectant approach, one‐step conservative surgery and the Triple P procedure, have been developed to address the desire for fertility preservation and reduce surgical complications. Due to limited data in the field, current guidelines do not offer definitive recommendations for the most appropriate management approach in specific clinical situations. Therefore, the management of PAS disorders requires an individualised approach based on various factors, such as the extent of placental invasion, topography, ability to achieve haemostatic control, the patient’s desire for future fertility, and the available medical resources and surgical expertise. The current literature review explores the efficacy and safety of both nonconservative and conservative management strategies, highlighting their impacts on maternal and neonatal outcomes, surgical morbidity and future fertility potential.

## 1. Introduction

**Figure 1:**
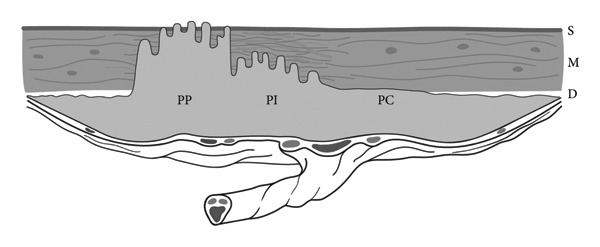
Placenta accreta spectrum. Placenta creta (PC) is characterised by adherence to the decidual layer (D) without invasion into the myometrium (M), placenta increta (PI) is characterised by physical invasion of villi into the myometrium, and placenta percreta (PP) is characterised by full‐thickness invasion of embryonic villi through the myometrium and its serosa (S). Taken with permission from Jauniaux et al. Pathophysiology and Ultrasound Imaging of Placenta Accreta Spectrum. American Journal of Obstetrics and Gynecology 2018 [1].

Placenta accreta refers to a spectrum of placental abnormalities in which the embryonic villi pathologically adhere to the myometrium or invade it [1]. The continuous spectrum can be categorised into three distinct grades (Figure 1): placenta creta (80%) (often referred to as placenta ‘accreta’ in the literature), placenta increta (15%) and placenta percreta (5%) [1, 2]. Current pathophysiological hypotheses of placenta accreta spectrum (PAS) disorders support the concept of secondary defects in the endometrium‐myometrial interface, which are believed to lead to failure in normal decidualisation, thus promoting deep trophoblast infiltration and villi anchoring, resulting in accretism. As such, the predisposition of PAS can be attributed to iatrogenic factors related to surgical damage, such as caesarean section (CS), in up to 95% of cases [3]. Other recognised risk factors include myomectomy, hysteroscopy, IVF procedures, endometrial resection, surgical termination of pregnancy, radiation, adenomyosis and submucous fibroids, amongst others [4].

Obstetric procedures in patients with PAS carry significantly higher risks compared to those without PAS. In 2021, Matsuzaki et al. conducted a large retrospective study in the United States comparing outcomes of caesarean deliveries in 8030 PAS patients and 2,719,447 non‐PAS patients [5]. Their findings showed that PAS patients were significantly more likely to undergo hysterectomy (53.5% vs. 0.4%, *p* < 0.001), cystoscopy (5.6% vs. 0.1%, *p* < 0.001) and oophorectomy (4.6% vs. 0.5%, *p* < 0.001) during caesarean delivery. PAS patients also had a markedly higher risk of cardiac arrest and rhythm conversion (0.4% vs. < 0.1% for both, *p* < 0.001), venous thromboembolism (0.2% vs. < 0.1%, *p* < 0.001) and shock (5.1% vs. < 0.1%, *p* < 0.001). Furthermore, PAS patients faced a sixfold increased risk of acute renal failure and an eighteenfold increased risk of acute respiratory syndrome, both of which can be seen in the setting of hypovolaemic shock and massive transfusion.

The management of PAS disorders presents a complex scenario in obstetric care, with potential life‐threatening complications for both mother and foetus. Traditionally, the standard approach has been caesarean hysterectomy, in which the uterus is removed with the placenta left in situ [6]. However, with evolving evidence and ongoing discussions, there has been a growing exploration of uterine‐conserving surgical methods aimed at preserving fertility and minimising maternal morbidity. The current review serves as a comprehensive exploration of the evolving landscape of PAS disorder surgical management, highlighting the pivotal role of both conservative and nonconservative approaches in ensuring the most favourable outcomes for patients with PAS disorders.

### 1.1. Classification of PAS Disorders

Recent research indicates that up to 50% of PAS cases remain undiagnosed prenatally [7]. Correct diagnosis or a high index of clinical suspicion during the antenatal period is critical, as it facilitates a multidisciplinary approach to care. This, in turn, has been shown to reduce intrapartum blood loss, the need for blood product transfusion and overall maternal morbidity by 50% [8]. Given the known risk factors for PAS, it is imperative that all pregnant patients with previous uterine surgery or low‐lying anterior placenta undergo expert evaluation of the myometrial–placental interface no later than 18–24 weeks [1]. Notably, as certain radiological markers of PAS can manifest as early as the first trimester, some experts advocate for assessment as early as 11–14 weeks [9] (Jauniaux et al., 2023). Ultrasound serves as the gold standard modality for routine antenatal screening and diagnosis of PAS disorders, with reported sensitivity and specificity up to 90%. However, its diagnostic accuracy heavily relies on the examiner’s expertise and training [8]. However, there is considerable heterogeneity in diagnostic protocols across centres, which affects the universal reporting system of PAS disorders and impacts the quality of data in international literature.

Magnetic resonance imaging (MRI) has been employed as an adjunct to ultrasound, offering a broader field of view and superior soft tissue resolution. This is particularly beneficial in detecting adjacent organ invasion, where ultrasound may be limited by the depth of accretion or interference from foetal parts. Noncontrast MRI performed between 24 and 30 weeks of gestation has proven valuable for assessing posterior placenta praevia, bladder invasion and the myometrium–placenta interface in patients with previous hysterotomy [8]. While ultrasound remains the gold standard, adjuvant MRI is recommended by both the FIGO consensus and the Royal College of Obstetricians and Gynaecologists (RCOG) guidelines for diagnostic validation and for assessing the depth and topography of placental invasion [1]. The widespread adoption of these imaging modalities has improved diagnostic accuracy and highlighted the importance of individualised, multidisciplinary management strategies.

As will be discussed, hysterectomy is often regarded as the standard treatment for most PAS cases, irrespective of severity or potential clinical outcomes. This is largely attributable to the difficulties of implementing an individualised approach, leading many tertiary centres to establish standardised management protocols and to train staff specifically in performing peripartum hysterectomy. Consequently, hysterectomy often becomes the default management strategy of PAS in these institutions. However, over the past decade, there has been a gradual yet significant shift towards more individualised management, challenging this uniform, one‐size‐fits‐all approach. Within this evolving landscape, increasing interest has emerged in the topographical classification of placental invasion as a means of guiding tailored treatment strategies. This approach, championed by a group of experts in Argentina, holds promise in refining the management of PAS and promoting a more nuanced, patient‐centred approach [10].

It is widely acknowledged that preoperative radiological classification should be complemented by postoperative histological analysis, which plays an important role in the retrospective diagnosis and assessment of individual PAS cases [11]. However, this cannot inform intraoperative decision‐making. In contrast, topographical classification is increasingly recognised as having a strong correlation with the surgical complexity of PAS management, as well as the severity of maternal morbidity and mortality [10]. This classification system aims to define the specific uterine regions affected by abnormal placental invasion (anterior, posterior, or lateral), the relationship of the lesions to the peritoneal reflection (above or below), and the histopathological characteristics of the accreted placenta (e.g., neovascularisation or fibrosis involving surrounding viscera) [10]. It is based on the principle that each potentially affected uterine region is anatomically linked to distinct arterial pedicles and neighbouring structures, including components of the urinary and vascular systems [10]. Therefore, each topographical class corresponds to a conceptual three‐dimensional anatomical model, which can be used to guide the selection of the most appropriate surgical technique and adjunct vascular procedures [10]. Importantly, this classification can be applied intraoperatively during caesarean laparotomy. Once safe dissection of the involved uterine areas is achieved, the surgeon can assess the topography of invasion and select the most appropriate surgical approach accordingly [10].

**Figure 2:**
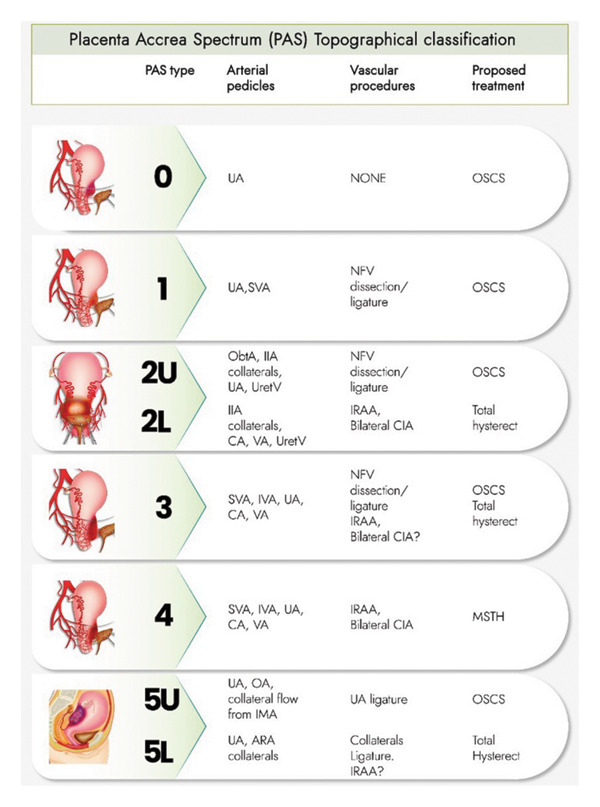
Intraoperative topographic classification of PAS and proposed treatments. Type 0 involves a uterine ‘window’ or dehiscence. Type 1 entails involvement of the upper part of the uterine segment. Type 2 is characterised by parametrial involvement, with subcategories 2U (upper parametrial involvement) and 2L (lower parametrial involvement). Type 3 encompasses involvement of the cervix or the lower part of the uterine segment, situated below the peritoneal reflection. Type 4 comprises the features of type 3 alongside vesicouterine fibrosis. Type 5 involves the posterior wall of the uterus, with subdivisions 5U (upper part of the wall involvement) and 5L (below the peritoneal reflection level). ARA, anterior rectal artery; CA, cervical artery; CIA, common iliac artery; IIA, internal iliac artery; IMA, inferior mesenteric artery; IRAA, inferior aortic artery; IVA, inferior vesical artery; MSTH, modified subtotal hysterectomy; NVF, newly formed vessels; OA, ovarian artery; ObtA, obturator artery; OSCS, one‐step conservative surgery; SVA, superior vesical artery; UA, uterine artery; UretV, ureteral vessels; VA, vaginal arteries. Taken with permission from Palacios‐Jaraquemada, et al. 2023. Advantages of Individualising the Placenta Accreta Spectrum Management. Frontiers in Reproductive Health, 4, p.1096175 [10].

Palacios et al. have described several topographical subtypes of PAS (Figure 2) [10]: Type 0 is characterised by a uterine ‘window’ or dehiscence, while Type 1 involves the upper uterine segment. Type 2 is defined by parametrial involvement and is further subdivided into 2U (upper parametrial involvement) and 2L (lower parametrial involvement). Type 3 involves the cervix or lower uterine segment, below the peritoneal reflection. Type 4 shares the same features as Type 3 but also includes vesicouterine fibrosis. Type 5 involves the posterior uterine wall and is further categorised into 5U (upper wall involvement) and 5L (below the peritoneal reflection). Each of these types correlates with varying degrees of surgical complexity and necessitates distinct management strategies. This highlights the importance of both preoperative understanding of anatomical involvement and intraoperative topographical assessment in achieving balanced and individualised PAS care. The proposed surgical approaches for each PAS subtype, whether radical or conservative, will be explored in further detail in the following sections.

## 2. Nonconservative Surgical Management

Currently, the most widely accepted management approach for patients with PAS involves performing a caesarean hysterectomy with the adherent placenta left in situ. Indeed, up to 89.3% of PAS cases diagnosed antenatally undergo either elective or emergency caesarean hysterectomy. The decision to perform a caesarean hysterectomy is often made preoperatively, particularly when there is a high suspicion of accretism and no desire for future fertility. Total caesarean hysterectomy is generally preferred, as it obviates the need for ongoing cervical screening and reduces the potential risk of cervical stump malignancy, as well as other complications such as abnormal discharge or bleeding [6]. Furthermore, subtotal hysterectomy is generally considered ineffective in managing cases of placenta increta or percreta with cervical involvement [6].

An alternative ‘definitive’ nonconservative surgical management strategy for PAS disorders is delayed hysterectomy [6]. This approach allows for partial placental resorption, reduced vascularity and uterine involution, thereby potentially facilitating subsequent surgical intervention [6]. Delayed hysterectomies are typically performed between 3 and 12 weeks postpartum and may involve postdelivery procedures such as internal iliac artery ligation or uterine artery embolisation [12]. In addition, delayed hysterectomy may serve as a safety net where complications arise during conservative management (e.g., expectant approach), as will be discussed later. However, it is crucial to acknowledge the inherent risks of this approach. Persistence of trophoblastic tissue can drive the consumption of coagulation factors, particularly fibrinogen, thereby increasing the risk of coagulopathies such as DIC and subsequent massive haemorrhage [13]. In emergency situations, leaving the placenta in situ may be the most appropriate interim measure, particularly in level 2 care facilities, where stabilisation is required prior to transfer to a specialised PAS centre.

### 2.1. Nonconservative Surgical Management Outcomes

One of the primary complications associated with nonconservative management of PAS is substantial blood loss, frequently estimated at an additional 2–3 L, with reported average estimated blood loss (EBL) reaching 3–5 L [8]. This extensive blood loss necessitates blood transfusions in up to 90% of cases, highlighting the critical importance of comprehensive preoperative planning [8]. In cases of deeply invasive PAS, there is a high risk of injury to major blood vessels and adjacent organs, such as the bladder and intestines, during attempts to separate the placenta from the uterine wall, thereby further complicating surgical outcomes [5]. Hysterectomy procedures performed for PAS are also associated with a higher incidence of postoperative complications, including infection and prolonged hospitalisation, underscoring the need for rigorous postoperative monitoring and care [5]. While hysterectomy effectively mitigates the risk of life‐threatening haemorrhage, it inevitably results in irreversible infertility, with profound implications for the patient’s reproductive future and psychological well‐being. Allen et al. [5] provide a detailed summary of these surgical complications in cases managed by caesarean hysterectomy (Table 1).

**Table 1:** Surgical complications associated with caesarean hysterectomy for PAS disorders.

**Complications**	

Median estimation of blood loss	2–3 L
Median units of packed red blood cells transfused	3.5–5.4 L
Large‐volume blood transfusion (> 10 L)	5%–40%
Injury to bladder	7%–48%
Injury to ureter	0%–18%
Admission to the intensive care unit	15%–66%
Bowel injury/obstruction	2%–4%
Venous thromboembolism	4%
Surgical site infection	18%–32%
Reoperation	4%–18%
Maternal mortality	1%–7%

*Note:* Taken with permission from Allen et al., FIGO Consensus Guidelines on Placenta Accreta Spectrum Disorders: Nonconservative Surgical Management. International Journal of Gynecology & Obstetrics 2018 [6].

When comparing clinical outcomes of subtotal versus total hysterectomy, in one study subtotal hysterectomy was associated with a reduced requirement for blood transfusions compared to total hysterectomy (32% vs. 83%, *p* > 0.02) [14]. However, another study found no significant difference in total blood loss between the two procedures (2.6L vs. 2.8L, *p* = 0.349) [15]. Similarly, no advantage has been demonstrated for subtotal hysterectomy in terms of operating times (142 vs. 136 min, *p* > 0.05) [14] or reduction in postoperative complications [14]. Findings regarding urological injury were also inconsistent. One study reported a lower rate of bladder injury with subtotal hysterectomy (40% vs. 12.5%, *p* < 0.001) [15], while the other found no significant difference between the two procedures (0% vs. 20%, *p* = 0.23) [14]. Furthermore, no significant differences have been observed in the frequency of bowel symptoms or sexual function between subtotal and total hysterectomy [16].

Intuitively, surgical morbidity and mortality are strongly correlated with the depth of placental invasion. For example, patients with placenta percreta are significantly more likely to require blood products, intensive care and sustained urologic injury compared to those with lower accretism grades [8]. A recent U.S. study analysing 7864 PAS‐related hysterectomy cases reported an all‐cause readmission rate of 7.3% within 60 days, with most readmissions occurring within the first 10 days postdischarge [17]. The most common diagnoses at readmission were infection (35%), thromboembolism (10%), hypertensive disorders (7%) and wound complications (5%). The inpatient mortality rate was significantly higher in the increta/percreta group, with an OR of 13.23 (95% CI 3.35–52.3) [17]. The study also compared operative outcomes across placenta accreta, increta and percreta (Table 2).

**Table 2:** Hysterectomy outcomes associated with specific PAS subtypes.

Placentation	Accreta (*n* = 5236)		Increta/percreta (*n* = 2628)			
Outcomes	*N*	%	*N*	%	OR (95% CI)	aOR (95% CI)
Nontransfusion severe maternal morbidity	895	17.1%	616	23.5%	1.49 (1.26–1.76)	1.53 (1.29–1.82)
Thromboembolism	76	1.5%	46	1.76%	1.18 (0.66–2.09)	1.16 (0.65–2.06)
Intraoperative complications	704	13.4%	710	27%	2.40 (2.02–2.85)	2.45 (2.05–2.93)
Reoperation	88	1.7%	68	2.6%	1.57 (1.0–2.44)	1.58 (1.0–2.45)
Surgical site complications	442	8.5%	340	12.9%	1.64 (1.31–2.04)	1.64 (1.31–2.06)
Haemorrhage	3491	46.0%	1084	41.2%	0.82 (0.71–0.94)	0.9 (0.78–1.05)
Sepsis	453	8.7%	348	13.2%	1.62 (1.29–2.03)	1.54 (1.22–19.6)
Inpatient mortality	DS	DS	DS	DS	13.23 (3.35–52.3)	NA

*Note:* DS, data suppressed because of cell count ≤ 10 for maternal mortality. Taken with permission from Overton et al. Outcomes Associated with Peripartum Hysterectomy in the Setting of Placenta Accreta Spectrum Disorder. American Journal of Obstetrics and Gynecology 2023 [17].

Abbreviations: aOR, adjusted odds ratios; NA, not applicable; OR, odds ratio.

A retrospective study involving 34 patients demonstrated that delayed hysterectomy was associated with significantly lower total EBL compared to immediate hysterectomy (1300 mL vs. 3000 mL, *p* < 0.01) and reduced the need for blood transfusions (median of 0 vs. 4 units, *p* = 0.016) [18]. Similarly, another study reported a reduction in EBL from 2800 mL during caesarean hysterectomy to 1650 mL in cases managed with CS followed by delayed hysterectomy (*p* = 0.01) [19]. The requirements for blood transfusions were also lower in the delayed hysterectomy group (3 vs. 6 units, *p* = 0.049) [20]. Furthermore, a linear regression analysis examining outcomes from 0 to 90 days postsurgery found that longer intervals between CS and hysterectomy were associated with a significant reduction in blood transfusion requirements (*p* = 0.019) and showed a trend toward lower EBL (*p* = 0.065) [20]. Despite these potential benefits, delayed hysterectomy carries notable interval risks, including coagulopathy, haemorrhage, fistula formation and sepsis [6]. Accordingly, the FIGO guidelines suggest that delayed hysterectomy may be considered in cases of placenta percreta with extensive pelvic invasion, where the benefits in blood loss reduction and transfusion requirements may outweigh the interval risks [6].

Finally, regarding urinary tract injury related to PAS hysterectomies, a systematic review including 289 cases from 49 publications reported an overall rate of iatrogenic urinary tract injury of 29%, with the bladder involved in 78% of cases and the ureter in 17% [21]. Stratified by PAS subtype, the incidence of urinary tract injury was highest in placenta percreta (24.5%) cases, followed by increta (11.8%) and accreta (5.2%), compared to 0.2% in non‐PAS cases [5]. Overall, 6.0% of all patients with accretism required either partial or total urinary cystectomy [5]. Although prophylactic use of preoperative cystoscopy and ureteric stent placement has been proposed to reduce the risk of urologic injury [21], a recent systematic review and meta‐analysis showed no significant benefits with these interventions (26.4% vs. 25.5%, RR = 0.94, 95% CI = 0.74–1.20) [22].

The main limitations of the current literature on outcomes of PAS‐related hysterectomy procedures lie in the retrospective nature of most studies, the lack of standardised preoperative staging, and the absence of information regarding whether the choice of surgical technique was influenced by surgeon preference, patient preference, or anatomical considerations based on lesion topography. Emerging evidence suggests that outcomes may be improved when the surgical approach is selected based on comprehensive preoperative evaluation and intraoperative topographical assessment.

## 3. Conservative Surgical Management

The conservative approach to the management of PAS aims to preserve fertility while minimising the psychosocial impact of hysterectomy, including effects on the patient’s social standing and self‐esteem. This strategy typically involves surgical procedures in which the placenta is left partially or entirely in situ, avoiding active removal. The retained placental tissue may subsequently undergo spontaneous resorption or be managed through localised resection of focal areas of accretism.

Several experts suggest that individuals with placental invasion located superiorly to the trigone of the bladder may be suitable candidates for conservative surgery, providing there is adequate vascular control over the uterine, upper vaginal and vesical arteries [23, 24]. Notably, predictions by Palacios et al. [23] indicate that up to 80% of PAS cases involve the superior and posterior bladder segment and spare the trigone, suggesting that hysterectomy could potentially be avoided in the majority of cases. A recent systematic review and meta‐analysis by Pan et al. [25] demonstrated that conservative management, compared to caesarean hysterectomy, was associated with significantly better outcomes. Specifically, conservative management resulted in lower EBL (WMD −1623.83 mL; 95% CI: −2337.87, −909.79; *I* ^2^ = 91.20%), reduced transfusion requirements for both packed red blood cells (WMD −2.37 units; 95% CI: −3.70, −1.04; *I* ^2^ = 86.61%) and fresh frozen plasma (WMD −0.40 units; 95% CI: −0.62, −0.19; *I* ^2^ = 0.00%). Additionally, conservative management was associated with a lower risk of bladder injury (RR 0.24; 95% CI: 0.11, 0.50; *I* ^2^ = 0.00%), coagulopathy (RR 0.20; 95% CI: 0.06, 0.74; *I* ^2^ = 0.00%) [25], and ICU admission (RR 0.24; 95% CI: 0.11, 0.52; *I* ^2^ = 0.00%) and had shorter operating times (WMD −73.69 min; 95% CI: −90.52, −56.86; *I* ^2^ = 24.07%) [25]. However, conservative management was associated with higher risk of endometritis (RR 10.91; 95% CI: 1.36, 87.59; *I* ^2^ = 0.00%) and hospital readmission (RR 8.99; 95% CI: 4.00, 12.21; *I* ^2^ = 0.00%) [25]. Other studies have also shown that PAS managed conservatively increases the risk of other future obstetric complications, including recurrent PAS, postpartum haemorrhage (PPH), uterine rupture, and peripartum hysterectomy [26, 27].

The international literature describes three main conservative methods, including the expectant approach, one‐step conservative surgery (OSCS), and the Triple P procedure. In contrast, the extirpative method, which involves forceful removal of the placenta to evacuate the uterus, is now largely discouraged due to its high risk of severe haemorrhage and adjacent organ injury. It will be discussed only briefly for completeness.

### 3.1. Extirpative Technique

The extirpative technique was historically used to reduce the risk of PPH by removing retained placental tissue [28]. However, in cases of PAS, this approach is unequivocally contraindicated. Even with meticulous surgical planning, manual removal of an abnormally adherent placenta significantly increases the risk of catastrophic haemorrhage and the need for emergency hysterectomy. Such haemorrhage may be compounded by coagulopathy, including disseminated intravascular coagulation (DIC), further escalating maternal morbidity and, in some cases, mortality [29]. Multiple guidelines, including those from FIGO, strongly advise against manual removal of the placenta in cases of suspected PAS. Attempts should be avoided if any unexplained difficulty is encountered during placenta delivery [6]. If placental removal is deemed absolutely necessary, it must be performed within a framework of careful surgical planning, with particular attention to vascular anatomy. As previously emphasised, effective vascular control is dependent on thorough preoperative and intraoperative topographical assessment, which should guide any decision to proceed with placental removal.

### 3.2. Expectant Approach

The expectant approach involves retaining the invasive placenta in situ with the aim of allowing spontaneous resorption over time [30]. This method is based on the physiological principle that, following delivery, uterine blood flow progressively decreases, thereby reducing perfusion to the parametrium and placenta. This diminished circulation leads to secondary villus necrosis and, theoretically, gradual placental detachment and sloughing [30]. The entire process, including resorption of any residual villous tissue, may take up to six months [30]. Adjuvant interventions, such as vessel ligation, uterine artery embolisation, or internal iliac balloon occlusion, can also be employed to expedite placental resorption and minimise morbidity. However, rates of resorption and expulsion vary considerably among individuals [8]. The expectant approach may be suitable for haemodynamically stable patients postdelivery, particularly when hysterectomy carries a high risk of incontrollable haemorrhage, or when practised in lower‐level institutions where it may enable safe transfer to a higher‐level facility for definitive management. However, in high‐risk cases, such as those with involvement of the bladder trigone, parametrium, cervix, or major pelvic vessels, the expectation that leaving the placenta in situ will not result in serious complications (e.g., coagulopathy, sepsis) may be overly optimistic. The limited evidence supporting this approach, along with the high risk of complications, raises questions about its widespread applicability. Although some guidelines recommend prophylactic use of broad‐spectrum antibiotics or methotrexate, the RCOG advises against their routine use due to insufficient evidence of benefit [7, 8].

#### 3.2.1. Expectant Approach Outcomes

In the largest retrospective study conducted to date, involving 167 cases of expectant management, 78% achieved ‘successful’ outcomes, defined as uterine preservation [27]. Among these patients, spontaneous placental resorption occurred between 4 and 60 weeks postdelivery, with a median duration of 13.5 weeks. However, 65% required subsequent pelvic devascularisation via procedures such as uterine artery embolisation and hypogastric artery ligation. Despite these promising results, some experts have questioned the study’s validity, noting that up to 50% of patients lacked an antenatal radiological diagnosis of PAS, thereby introducing potential bias [31, 32]. Indeed, other systematic reviews have reported lower success rates for expectant management, ranging from 42% [31], 58% [33] and 61.8% [34]. Encouragingly, the potential for future fertility remains high, with 83%–89% of patients desiring future pregnancy conceiving spontaneously following expectant management [27, 35, 36]. Nonetheless, this approach carries substantial risks. Early complications include infection, PPH, need for emergency hysterectomy, and maternal death [8]. Delayed complications include secondary amenorrhoea and intrauterine synechiae [8]. A study by Clausen et al. [33] found that 42% of patients managed expectantly experienced major complications, including sepsis, DIC, pulmonary embolisms, fistulae and arteriovenous malformations. Furthermore, 58% of patients eventually required delayed/secondary hysterectomy. By contrast, Sentilhes et al. reported a lower hysterectomy rate, either primary or secondary, of 21.6% [27]. Importantly, the risk of recurrent PAS in subsequent pregnancies was estimated at 22%–29%, a significant consideration when counselling patients on the long‐term implications of expectant management [8, 27].

### 3.3. OSCS

Another popular conservative approach is the OSCS. This approach involves surgical excision of the invasive portion of the placenta, followed by prompt reconstruction of the uterus using adjacent myometrial tissue and reinforcement of the bladder, when required [37].

Patient selection for OSCS is critical, and suitability is generally determined based on three anatomical criteria: (1) the uterus can be separated from the bladder without vesicouterine fibrosis; (2) there is a minimum of 2 cm of healthy myometrium above the cervix; and (3) the extent of the pathological myometrial involvement does not exceed 50% of the axial circumference of the uterus (Figure 3) [38]. If these criteria are not met, uterine reconstruction becomes technically difficult or unfeasible, and total hysterectomy is often considered the safer alternative, though this decision should be made on an individual basis [38]. Attempting OSCS in patients who do not meet these criteria may significantly increase the risk of uterine ischaemia, infection and necrosis [8]. However, it is crucial to acknowledge that the majority of PAS cases are not severe. As demonstrated here, individualised management guided by thorough topographical assessment allows for more tailored and potentially conservative treatment strategies, rather than adopting a uniform approach for all patients.

Figure 3Accretism assessment. (a) Adequate myometrial tissue lies between the PAS area and the cervix in the frontal (F) view. Lateral (L) and axial views (Ax) show that > 50% of the uterine circumference remains unaffected, suggesting suitability for OSCS. (b) Absence of healthy myometrium above the cervix, with both lateral and axial views indicating less than 50% of the uterine circumference being unaffected, suggesting unsuitability for OSCS. Taken with permission from Nieto‐Calvache et al. How to Perform the One‐Step Conservative Surgery for Placenta Accreta Spectrum Move by Move. American Journal of Obstetrics and gynecology 2023 [38].

**Figure:**
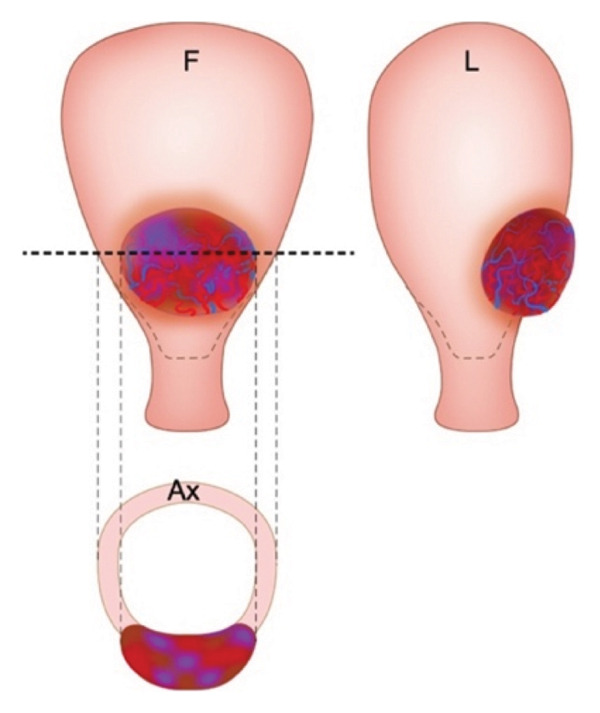
(a)

**Figure:**
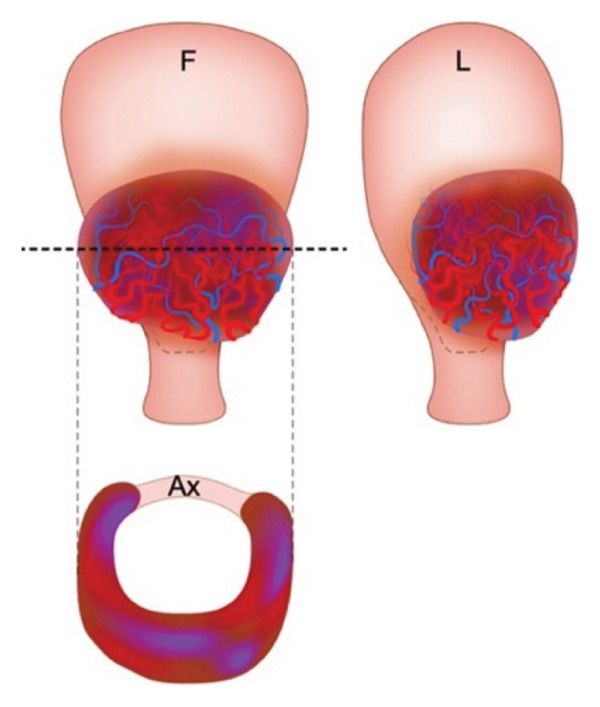
(b)

#### 3.3.1. OSCS Outcomes

In a study involving 68 individuals diagnosed with anterior placenta percreta, uterine preservation was successfully achieved in 50 cases (73.5%), although outcomes varied significantly depending on the level of bladder invasion: 95.7% preservation in cases with upper bladder invasion, compared to 27.3% with lower bladder invasion [39]. Similarly, another study found that the effectiveness of the procedure varied across different topographical PAS types: 81.5% in type 1, characterised by invasion into the upper posterior bladder; 47.7% in type 2, involving parametrial invasion; 21.8% in type 3, with low posterior bladder involvement; and 0% in type 4, featuring low posterior bladder involvement and associated fibrosis [40]. Another recent retrospective analysis, involving 202 patients undergoing OSCS, revealed that maternal and neonatal outcomes in subsequent pregnancies were comparable to those of uncomplicated pregnancies or pregnancies complicated by CS [23, 41]. Additionally, in a retrospective study by Sentilhes et al. involving 131 PAS patients, 88.9% of patients who desired future fertility had successful pregnancies, with a mean time to conception of 17.3 months [42]. However, intrauterine adhesions and associated secondary amenorrhoea were reported in 8.3% of cases, potentially compromising fertility [21].

When comparing clinical outcomes of OSCS and caesarean hysterectomy, an RCT of 91 patients reported no significant differences in median intraoperative blood loss (1500 vs. 1740 mL, OR = 1, *p* = 0.942), surgery duration (135 vs. 155 min, OR = 0.99, *p* = 0.151), transfusion rates (58.6% vs. 61.3%, OR = 0.96, *p* = 0.768) and overall adverse events (17.2% vs. 9.7%, OR = 1.77, *p* = 0.398) [43]. Another prospective study of 75 PAS patients similarly showed no significant difference in blood transfusion requirements (2 units for both, *p* = 0.288) or urinary tract injury rates between OSCS and hysterectomy groups [38, 43, 44]. However, OSCS patients required fewer vascular interventions than hysterectomy patients (4.69% vs. 27.27%, *p* = 0.011) [38]. Neonatal outcomes were also comparable. A retrospective review of 296 PAS cases found no significant differences between OSCS and hysterectomy groups in 1‐ and 5‐min APGAR scores (5 vs. 5, *p* = 0.747; 7 vs. 7, *p* = 0.761), oxygen support requirements (51.6% vs. 49.4%, *p* = 0.732), NICU admission and duration (43.3% vs. 45.6%, *p* = 0.73; 6 vs. 7 days, *p* = 0.646), and major neonatal complications (1.8% vs. 2.5%, *p* = 0.659) [45]. Another retrospective study evaluating a modified OSCS procedure incorporating B‐Lynch sutures demonstrated significantly reduced EBL (1000 vs. 1500 mL, *p* < 0.001), red blood cell transfusion volume (500 vs. 710 mL, *p* = 0.002) and incidence of visceral injuries (4.60% vs. 21.51%, *p* < 0.001) compared to caesarean hysterectomy [44]. However, the rate of postoperative infection was higher in the modified OSCS group (10.14% vs. 1.27%, *p* = 0.012). This can be attributed to uterine conservation, which can act as a focus for infection. Taken together, while the OSCS does not seem to significantly outperform hysterectomy in terms of immediate surgical outcomes, it represents a viable and safe fertility‐preserving alternative in appropriately selected patients, without increasing maternal or neonatal risk.

### 3.4. Triple P Procedure

**Figure 4:**
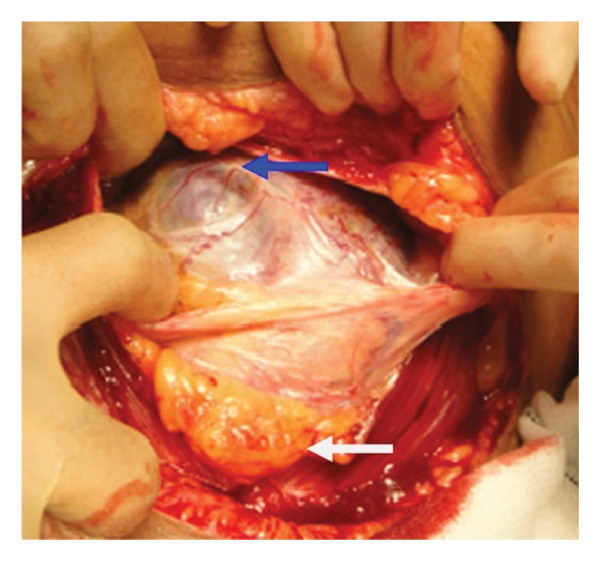
Positioning of the myometrial incision above the upper placental border. The blue arrow indicates the upper border of the placenta, which is protruding through the serosa. The white arrow highlights the lower uterine segment, where the placenta has invaded the urinary bladder. Additionally, the omentum is adherent to the area of uterine perforation at the site of a prior caesarean scar from earlier in the pregnancy. Taken with permission from Chandraharan et al., 2012. The Triple‐P Procedure as a Conservative Surgical Alternative to Peripartum Hysterectomy for Placenta Percreta. International Journal of Gynecology & Obstetrics, 117 (2), pp.191–194 [46].

The Triple P procedure, first introduced by Chandraharan in 2010 [46], comprises three main steps: (1) perioperative placental localisation and delivery of the foetus via a transverse uterine incision above the upper placental border (Figure 4); (2) pelvic devascularisation using bilateral internal iliac balloon occlusion and (3) nonseparation of the placenta with excision of the myometrium and reconstruction of the uterine wall (Figure 5). Although this may appear similar to the traditional ‘one‐step’ conservative approach, the Triple P procedure is distinct in that it combines upper placental border myometrial access with pelvic devascularisation to reduce intraoperative blood loss. It also includes intraoperative sonographic assessment of the placenta.

Figure 5Placental nonseparation and myometrial excision (a) resection of the myometrium beneath the placental bed, with the placenta remaining attached to the myometrial wall (blue arrow). The white arrow indicates the excised myometrium containing the morbidly adherent placenta following foetal delivery. The black arrow marks approximately 2 cm of myometrium above the bladder reflection, which is preserved to facilitate uterine closure. (b) Persistent placental adherence. The blue arrow highlights the excised myometrium, while the white arrow denotes the morbidly adherent placenta still attached to the remaining myometrial tissue. (c) Uterine appearance postmyometrial excision. The blue arrow identifies the superior lip of the uterine incision, which in this instance contains a fibroid. The white arrow points to the myometrial defect following excision. At the inferior margin of the incision (black arrow), maintaining at least 2 cm of myometrial tissue is crucial for effective closure. Additionally, prompt application of haemostatic clamps to the myometrial edges is essential to minimise excessive bleeding. Taken with permission from Chandraharan et al., 2012. The Triple‐P Procedure as a Conservative Surgical Alternative to Peripartum Hysterectomy for Placenta Percreta. International Journal of Gynecology & Obstetrics, 117 (2), pp. 191–194 [46].

**Figure:**
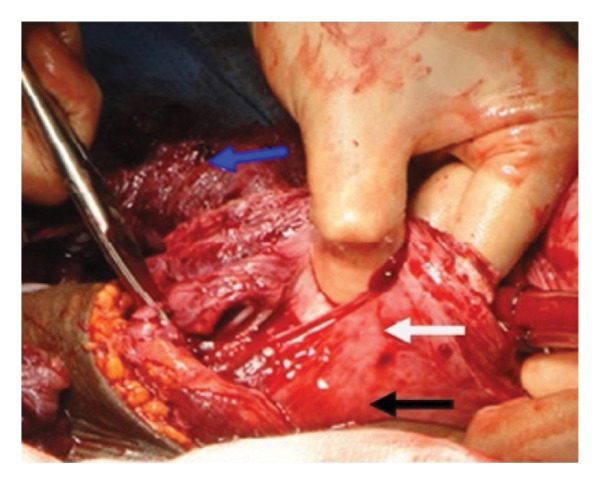
(a)

**Figure:**
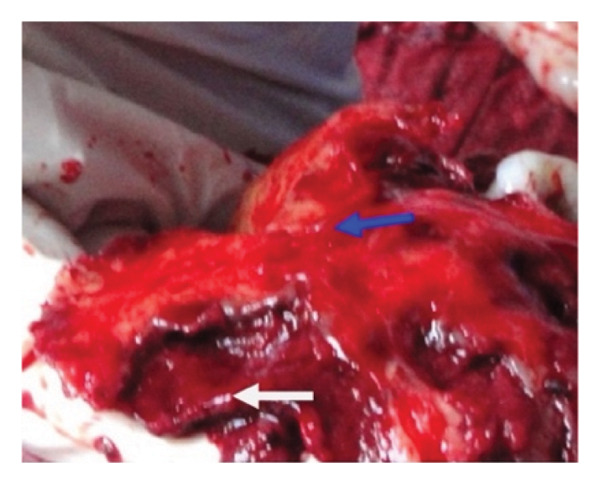
(b)

**Figure:**
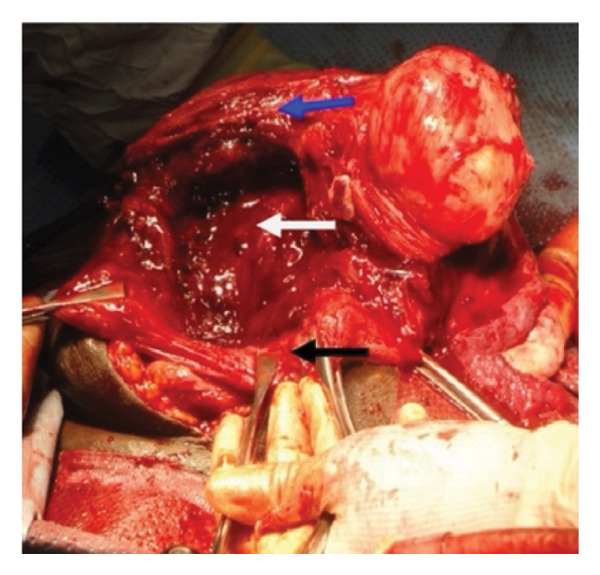
(c)

#### 3.4.1. Triple P Procedure Outcomes

A small‐scale study involving 30 patients compared the Triple P Procedure with the expectant approach, revealing a significantly reduced risk of PPH (15.8% vs. 54.5%, *p* = 0.035) [47]. A larger retrospective review of the initial 50 cases managed by Chandraharan’s group reported a mean blood loss of 2318 mL (400–7300 mL), with only 6% of patients requiring ITU admission for massive obstetric haemorrhage [48]. Urinary tract injury was low (2%), and no patients required immediate or delayed hysterectomy. The arterial thrombosis rate was also limited to 6%, with no reported long‐term sequelae.

Zhao et al. [49] proposed a modified Triple P procedure specifically targeting placenta percreta and intending to reduce intraoperative bleeding more effectively. Their adaptation combined single aortic balloon occlusion with a uterine tourniquet and an innovative haemostatic technique involving a continuous suture along the lower uterine incision. In a cohort of 142 patients, this method demonstrated a significantly lower mean EBL of 1200 mL (687–1812 mL), compared to 1300 mL (800–2500 mL) in a nonstandardised control group employing various other techniques (*p* = 0.04) [49]. Although the lack of a standardised procedure in the control group limits definitive conclusions, the findings support the modified Triple P as a potentially safer option for percreta cases. Complication rates remained low, with urinary tract injury in 2.1%, thrombosis in 2.8%, and no reported emergency hysterectomies. Reproductive outcomes were encouraging, with 95.3% of patients resuming menstruation within 10 months. Chronic pelvic or limb pain was rare, and intrauterine adhesions were observed in only 0.8% of cases. Collectively, these findings suggest that both the original and modified Triple P Procedure offer promising fertility‐preserving alternatives for selected PAS cases, particularly in the context of structured surgical protocols. However, most studies emphasised the need for adjunctive PPH control techniques, underscoring the importance of a multimodal approach. However, many protocols apply these interventions uniformly, without accounting for the wide heterogeneity in PAS presentations. Preoperative and intraoperative evaluation should remain central to decision‐making when considering conservative management is planned. Tailoring the use of interventions such as vascular occlusion to specific intraoperative findings, rather than using them indiscriminately, may reduce unnecessary risk. For instance, a study by Nieto‐Calvache et al. demonstrated that intraoperative staging protocols can effectively guide selective use of vascular control techniques such as REBOA, enhancing safety without over‐treatment [50]. This targeted, individualised approach stands in contrast to nondiscriminatory protocols and reinforces the need for case‐specific decision‐making in PAS management [50].

## 4. Discussion

It is essential to move away from a rigid or ‘totalitarian’ approach to treatment selection in the management of PAS. Management decisions must be guided by individual clinical scenarios, anatomical topography and institutional resources, rather than retrospective generalisations or a singular, fixed model. Intraoperative findings, particularly when guided by topographical classification, should play a central role in real‐time decision‐making, allowing clinicians to adapt strategies to each case. We therefore strongly advocate for wider adoption of intraoperative topographical classification as a standard component of PAS surgery. This approach facilitates tailored, case‐specific management and avoids the pitfalls of one‐size‐fits‐all strategies. Centres employing this methodology are encouraged to explicitly describe their classification systems and how they informed, and in some cases altered, the surgical course. Doing so not only promotes transparency and reproducibility but also enables comparative research.

Although all surgical interventions inherently carry risks such as infection or haemorrhage, evidence from multiple large series suggests that complication rates fall within expected ranges, regardless of whether a radical or conservative technique is used, particularly when procedures are performed by experienced multidisciplinary teams. This further underscores the need for a flexible, evidence‐informed strategy that avoids treatment bias and prioritises patient‐specific factors over dogmatic adherence to a particular model.

A notable limitation of many current studies is their retrospective design, which restricts the ability to reliably assess outcomes, especially those reported by patients. Future research should adopt prospective study designs, allowing for baseline and longitudinal assessments of both clinical and patient‐reported outcomes. Furthermore, despite the growing body of literature on PAS management, the field still lacks RCTs, which are critical for determining the relative efficacy and safety of different surgical strategies. RCTs would be particularly valuable in guiding two‐step decision‐making processes—such as first determining whether a conservative or radical approach is appropriate and subsequently selecting the most suitable conservative strategy. They are also crucial for evaluating optimal timing of delivery, effectiveness of interventional radiology techniques, and preventative measures against organ damage.

In addition to more rigorous study design, extended follow‐up periods are imperative to evaluate the long‐term outcomes of various management strategies. These include impacts on physical and psychological well‐being, future fertility and neonatal development. Particularly in conservatively managed cases, where uterine preservation is prioritised, long‐term data are essential to fully understand the implications for future pregnancies and overall maternal health.

## 5. Conclusion

In conclusion, the management of PAS disorders presents a multifaceted challenge that requires a nuanced and individualised approach tailored to each patient’s circumstances, desires for future fertility, and available resources. While traditional approaches like caesarean hysterectomy with the placenta left in situ remain widely practised, there is a growing interest in exploring conservative management strategies. These approaches hold promise in reducing major obstetric haemorrhage and preserving the uterus, but their long‐term efficacy and safety necessitate further investigation through well‐designed clinical trials. The choice of specific surgical strategies, whether conservative or nonconservative, should be informed by intraoperative topographical classification, a method that is increasingly supported by a growing body of literature demonstrating its potential to improve clinical outcomes.

As our understanding of PAS continues to evolve, the field must remain grounded in individualised, patient‐centred care. Broad adoption of topographical classification systems, improved methodological rigour through RCTs and prospective multicentre designs and a commitment to long‐term follow‐up will together enable safer, more effective and more tailored treatment pathways for patients with PAS.

## Consent

No written consent has been obtained from the patients as there is no patient‐identifiable data included in this review.

## Conflicts of Interest

The authors declare no conflicts of interest.

## Funding

No funding was received for this manuscript.

## Data Availability

Data sharing is not applicable to this article, as no datasets were generated or analysed during the current study.
